# Contemporary Incidence and Survival of Lung Neuroendocrine Neoplasms

**DOI:** 10.1001/jamanetworkopen.2025.35125

**Published:** 2025-10-02

**Authors:** Julie Hallet, Mathieu Rousseau, Elliot Wakeam, Sten Myrehaug, Léamarie Meloche-Dumas, Anna Gombay, Wing Chan, Simron Singh

**Affiliations:** 1 Department of Surgery, University of Toronto, Toronto, Ontario, Canada; 2 Susan Leslie Clinic for Neuroendocrine Tumors, Sunnybrook Health Sciences Centre, Toronto, Ontario, Canada; 3 Cancer Program, ICES, Toronto, Ontario, Canada; 4 Evaluative Clinical Sciences, Sunnybrook Research Institute, Toronto, Ontario, Canada; 5 Department of Surgery, McGill University, Montréal, Québec, Canada; 6 Department of Radiation Oncology, University of Toronto, Toronto, Ontario, Canada; 7 Department of Medicine, University of Toronto, Toronto, Ontario, Canada

## Abstract

**Importance:**

While the epidemiology of overall and gastrointestinal neuroendocrine neoplasms (NENs) has been reported, data specific to lung NENs remain scarce.

**Objective:**

To examine the incidence, overall survival (OS), and lung cancer–specific death for lung NENs.

**Design, Setting, and Participants:**

Population-based retrospective cohort study in Ontario, Canada, of adult patients with incident lung NENs from 2000 to 2020. Data were analyzed from July to December 2024.

**Main outcomes and measures:**

Yearly incidence rates of lung NENs. OS examined with Kaplan-Meier curves and Cox regression models. Lung cancer–specific deaths using cumulative incidence function and Fine-Gray models accounting for the competing risk of death from other causes.

**Results:**

Among 4479 total patients, the median (IQR) age at diagnosis was 67 (57-74) years, and 2521 (56.3%) were female; 2056 (45.9%) had typical neuroendocrine tumors (NET), 370 (8.3%) atypical NET, 998 (22.3%) large cell neuroendocrine carcinoma (NEC, including small cell and mixed NEC), and 1055 (23.6%) other NEC, as well as 1103 (24.6%) who presented as stage IV. The incidence of lung NENs increased 2.87-fold from 0.87 to 2.50 per 100 000 from 2000 to 2020. This rise in incidence was observed mostly for typical NET (from 0.51 to 1.09) and for stage I (0.68 to 1.18). With a median (IQR) follow-up of 34 (9-87) months, 5- and 10-year OS were 50% (95% CI, 49%-51%) and 40% (95% CI, 39%-41%) overall. Advancing age, lower socioeconomic status, type of lung NEN, and advancing stage were independently associated with inferior OS. Cumulative incidence of lung cancer–specific deaths was 41% (95% CI, 40%-42%) at 5 years and 46% (95% CI, 45%-47%) at 10 years. Advancing age, type of lung NEN, and increasing stage were independently associated with higher hazards of lung cancer–specific deaths. Lung cancer–specific deaths were exceeded by deaths from other causes starting 2 year after diagnosis for typical NET and 3 years after diagnosis for stage I disease.

**Conclusions and relevance:**

The incidence of lung NENs has increased over 20 years, mostly associated with stage I disease. Prolonged OS was observed after lung NEN diagnosis. Patients with typical lung NET and stage I disease were more likely to die of causes other than lung cancer after 1 and 3 years, respectively. These data are important to direct efforts in care, research, and patient counseling.

## Key Points

**Question:**

What are the contemporary incidence trends and survival outcomes for patients with lung neuroendocrine neoplasms (NENs)?

**Findings:**

In this cohort study of 4479 patients, the incidence of lung NENs increased 2.87-fold from 2000 to 2020, primarily due to typical neuroendocrine tumors (NETs) and stage I disease. Ten-year overall survival was 40%, varying by histology and stage; for typical NETs and stage I tumors, non–cancer-related mortality exceeded lung cancer death within 2 and 3 years, respectively.

**Meaning:**

These findings highlight the evolving epidemiology of lung NENs and suggest that tailoring surveillance and treatment strategies based on tumor subtype and risk of competing mortality are crucial.

## Teaser

This cohort study evaluates the incidence, overall survival, and lung cancer–specific death for lung neuroendocrine neoplasms.

## Introduction

While neuroendocrine neoplasms (NENs) are most commonly diagnosed in the gastroenteropancreatic (GEP) system, lung NENs represent over 20% of all NENs.^1,2^ The behavior of lung NENs varies widely, ranging from more indolent disease such as typical neuroendocrine tumors (NETs) to more aggressive forms like neuroendocrine carcinomas (NECs).^3,4^ Despite the clinical relevance of lung NENs, they remain an understudied and poorly understood malignant neoplasm.

Globally, the incidence of NENs has risen in recent decades.^1,2^ However, the rising incidence and outcomes of NENs have primarily been studied in the context of GEP-NENs, with limited research focusing specifically on lung NENs. Population-level data on lung NENs are sparse, with existing studies often aggregating lung NENs with other NEN types or using older datasets that may not reflect contemporary diagnostic and treatment practices.^1,2,5,6,7,8^ This limitation of lung-specific epidemiology NEN data are critical, as the management of lung NENs may differ from that of other NEN subtypes, and the development of care pathways, surveillance strategies, and new clinical trials for therapy ought to be informed by accurate epidemiologic data.

This study used a population-based approach to examine the epidemiologic characteristics of lung NENs. It specifically aimed to describe the incidence of lung NENs as well as the overall survival (OS) and lung cancer (LC)–specific mortality after lung NEN diagnosis.

## Methods

### Study Design

Through ICES (formerly known as the Institute for Clinical Evaluative Sciences), linked administrative health care datasets from the province of Ontario, Canada, were used to conduct a retrospective cohort study. ICES is a prescribed entity under Ontario’s Personal Health Information Protection Act (PHIPA). Projects that use data collected by ICES under section 45 of PHIPA and use no other data are exempt from review and do not require informed consent to be gathered. The use of the data in this project is authorized under section 45 and approved by ICES’ Privacy and Legal Office. Reporting followed the Reporting of Studies Conducted Using Observational Routinely-Collected Health Data (RECORD) statement.^9^

### Data Sources, Study Population, and Study Cohort

Datasets housed at ICES were linked using unique encoded identifiers and analyzed at ICES (eTable 1 in Supplement 1). Those datasets included the Ontario Cancer Registry (OCR), the Ontario Registrar General Death database (ORGD), and the Registered Persons Database (RPDB) which contains vital status and demographic data. From Ontario’s 13.5 million residents, adult (≥18 years old) patients with a new diagnosis of lung NEN from January 1, 2000, to December 31, 2020, were identified in the OCR with *International Statistical Classification of Diseases and Related Health Problems, Ninth Revision (ICD-9)* O.3 codes using a strategy previously reported by our team further restricted to lung tumor site using *ICD-9* O.3 topography codes C34* (eTable 2 in Supplement 1).

### Outcomes

Outcomes of interest were incidence of lung NENs, death from any cause following lung NEN diagnosis, and LC-specific death following lung NEN diagnosis. Patients were followed up until date of death, date of last contact, or end of study date on November 30, 2022, whichever came first. This allowed for a minimum of 23 months of follow-up for all patients.

The incidence of lung NEN was determined for the adult Ontario population for each study year. Data capture for stage at diagnosis started in 2010 and was complete until 2019 at the time of analyses; therefore, analyses stratified by that variable were restricted to lung NENs diagnosed from January 1, 2010, to December 31, 2019.

For survival analyses, time to death was calculated from date of diagnosis. For OS, time to death from any cause was computed. LC-specific death was defined as *ICD-9*, code 162 and death from other causes as other codes in the ORGD (eTable 3 in Supplement 1). The primary cause of death was defined as the antecedent cause of death when available or the immediate cause of death when antecedent causes were not captured. The cause of death in ORGD was available until December 31, 2017. Analyses for LC-specific death were restricted to the cohort of lung NENs diagnosed from January 1, 2000, to December 1, 2016, allowing for a minimum opportunity of 12 months of follow-up for all patients.

### Sociodemographic and Clinical Characteristics

Baseline characteristics were measured at the time of diagnosis. Age and sex assigned at birth were captured in the RPDB. Comorbidity burden was measured using the Elixhauser comorbidity index with the number of comorbidities (excluding cancer and metastases diagnoses) summed as a continuous variable as well as dichotomized using a cutoff of 4 or greater for high burden.^10^ Rural residence was defined according to the Rurality Index of Ontario.^11^ Socioeconomic status was assessed with material deprivation quintile.^12^ Lung NENs were subdivided according to histological type of tumor following the World Health Organization 2017 classification into typical NET and atypical, large cell, and other NEC, which included small cell NEC and mixed NEC (eTable 2 in Supplement 1).^3,4,13^ Stage at diagnosis was captured in the OCR as best stage at diagnosis using the tumor, node, and metastasis staging system by the American Joint Committee on Cancer, 8th edition.^14^ Stage data were captured starting as of 2010 and complete until the end of 2019, such that analyses including this variable are restricted to those years.

### Statistical Analysis

Baseline patient characteristics for the entire cohort were described using absolute number and percentage for categorical variables and median (IQR) for continuous variables. Outcomes analyses were conducted for the entire cohort as well as stratified by sex, age at diagnosis, type of lung NEN, and stage at diagnosis. Incidence was computed with the defined adult Ontario population as the denominator for each year and was reported as the incidence per 100 000 per year. OS was estimated using Kaplan-Meier methods with censoring at the date of last contact or end of study date. LC-specific death was estimated with cumulative incidence functions of events following lung NEN diagnosis, treating deaths from other causes as a competing risk.^15^

To explore the independent association of potential prognostic factors on the probability of OS and LC-specific death, we constructed multivariable models. Cox regression models were used for OS. Fine-Gray regression models accounting for the competing risk of death from other causes were used for LC-specific death.^16,17^ Potential prognostic factors were selected a priori based on clinical relevance and literature review. Additional models restricted to lung NENs diagnosed from 2010 to 2019 were constructed to assess the association between outcomes and stage at diagnosis, adjusted for the other selected prognostic factors. Finally, all models were adjusted for year of diagnosis. Results were reported as hazard ratios (HR) for Cox regression and subdistribution hazard ratios (sHR) for Fine-Gray regression, both with 95% CIs.

Missing data for key variables were explored. Data were missing for rural residency in 0.2% and material deprivation in 1.1% of the cohort. A complete case analysis was performed whereby patients with missing data were excluded for analyses using these variables. Statistical significance was set at a 2-sided *P* ≤ .05. All analyses were conducted using SAS Enterprise Guide, version 7.1 (SAS Institute) from July to December 2024.

## Results

During the study period, 4479 patients were diagnosed with lung NENs in Ontario. The characteristics of the diagnosed patients are presented in eTable 4 in Supplement 1. The median (IQR) age at diagnosis was 67 (57-74) years, and 2521 (56.3%) were female. The majority were typical NET (2056 patients [45.9%]), followed by large cell NEC (998 patients [22.3%]), other NEC (1055 patients [23.6%]), and atypical NET (370 patients [8.3%]). Among 2976 with stage data, (1103 patients [24.6%]) presented with stage IV.

### Incidence

The incidence of all lung NENs increased from 0.87 per 100 000 per year to 2.50 per 100 000 per year, representing a 2.87-fold increase (eFigure 1 in Supplement 1). This rise in incidence was observed mostly for typical NET (from 0.51 to 1.09) and for stage I (0.68 to 1.18). The yearly incidence is detailed in Table 1. Similar patterns were observed for male and female participants and in age groups over 60 years (eFigure 1C in Supplement 1). There was no increase in incidence observed for those aged between 18 and 49 years, and the increase was mild for those aged between 50 and 59 years. The incidence increased by 2.1-fold for typical NETs, but was smaller for other types of lung NENs (eFigure 1 in the Supplement 1). Looking at stage at diagnosis, the incidence increased 1.73-fold for stage I but was less pronounced for stages II to IV (eFigure 1 in Supplement 1).

**Table 1.** Yearly Incidence of Lung Neuroendocrine Neoplasms (NENs) Per 100 000 Per Year

Year	All lung NENs	Sex		Age at diagnosis, y					Histology type				Stage at diagnosis^a^			
		Female	Male	18-49	50-59	60-69	70-79	≥80	Typical NET	Atypical NET	Large cell NEC	Other NEC	I	II	III	IV
2000	0.87	0.87	0.87	0.27	1.42	1.97	2.58	2.44	0.51	0.01	0.00	0.35	NA	NA	NA	NA
2001	0.95	1.07	0.81	0.40	1.22	2.39	2.54	1.76	0.60	0.01	0.00	0.33	NA	NA	NA	NA
2002	0.83	1.01	0.64	0.34	0.76	1.70	3.34	1.67	0.50	0.10	0.04	0.19	NA	NA	NA	NA
2003	1.04	1.06	1.03	0.24	1.47	3.11	2.89	2.90	0.47	0.07	0.17	0.33	NA	NA	NA	NA
2004	1.29	1.29	1.29	0.51	1.67	3.21	3.97	1.50	0.76	0.10	0.27	0.16	NA	NA	NA	NA
2005	1.30	1.35	1.26	0.42	1.61	2.64	5.58	1.67	0.81	0.06	0.18	0.26	NA	NA	NA	NA
2006	1.23	1.35	1.09	0.37	0.84	2.92	5.67	2.50	0.64	0.09	0.15	0.34	NA	NA	NA	NA
2007	1.20	1.33	1.06	0.37	1.35	3.65	2.81	2.63	0.63	0.03	0.30	0.24	NA	NA	NA	NA
2008	1.42	1.55	1.28	0.30	1.66	4.06	4.76	2.76	0.68	0.13	0.30	0.32	NA	NA	NA	NA
2009	1.54	1.93	1.12	0.44	1.34	4.37	5.32	2.68	0.81	0.08	0.30	0.35	NA	NA	NA	NA
2010	1.96	1.90	2.02	0.42	2.00	5.27	6.77	4.19	0.72	0.15	0.54	0.55	0.68	0.18	0.17	0.82
2011	2.08	2.19	1.96	0.47	1.99	4.55	7.64	5.98	0.86	0.11	0.58	0.52	0.81	0.27	0.22	0.67
2012	2.22	2.21	2.22	0.43	2.96	4.74	6.84	5.39	0.92	0.18	0.64	0.47	0.98	0.14	0.19	0.73
2013	2.50	2.64	2.35	0.40	2.64	5.66	9.68	4.86	0.91	0.27	0.70	0.62	0.95	0.18	0.29	0.96
2014	2.53	2.67	2.37	0.55	2.14	5.53	9.00	6.30	1.07	0.21	0.62	0.62	0.98	0.28	0.35	0.85
2015	2.59	2.83	2.34	0.45	3.03	5.78	8.61	4.77	1.13	0.21	0.53	0.72	1.03	0.24	0.27	0.94
2016	2.61	2.87	2.33	0.63	2.34	5.03	9.03	6.31	1.17	0.32	0.62	0.50	1.09	0.23	0.38	0.78
2017	3.26	3.57	2.92	0.57	3.01	7.48	10.40	6.97	1.58	0.39	0.68	0.61	1.10	0.19	0.34	0.95
2018	2.84	3.37	2.28	0.38	2.40	6.27	9.56	7.06	1.33	0.25	0.69	0.58	1.20	0.24	0.30	0.87
2019	2.97	3.36	2.55	0.38	2.12	6.72	10.74	6.23	1.23	0.22	0.79	0.72	1.18	0.24	0.31	0.98
2020	2.50	2.74	2.25	0.36	2.38	5.35	8.45	4.86	1.09	0.18	0.66	0.57	NA	NA	NA	NA
Absolute difference	1.63	1.87	1.38	0.09	0.96	3.38	5.87	2.42	0.58	0.17	0.66	0.22	0.5	0.06	0.14	0.16
Difference, %	187	215	159	33	68	172	228	99	114	1700	1650	63	74	33	82	20

Abbreviations: NA, not applicable; NEC, neuroendocrine carcinoma; NET, neuroendocrine tumor.

^a^ Data restricted to 2010 to 2019 due to availability.

### Overall Survival

The median (IQR) follow-up was 34 (9-87) months, and 2058 patients (56%) died during the follow-up period. This varied by type of NEN, with a median (IQR) of 77 (72-81) months for typical NETs, 52 (43-59) months for atypical NETs, 12 (11-13) months for large cell NECs, and 8 (7-8) months for other NECs. The median (IQR) follow-up was 73 (68-77) months for stage I disease, 60 (55-67) months for stage II, 22 (18-26) months for stage III, and 7 (6-7) months for stage IV.

**Figure 1.**
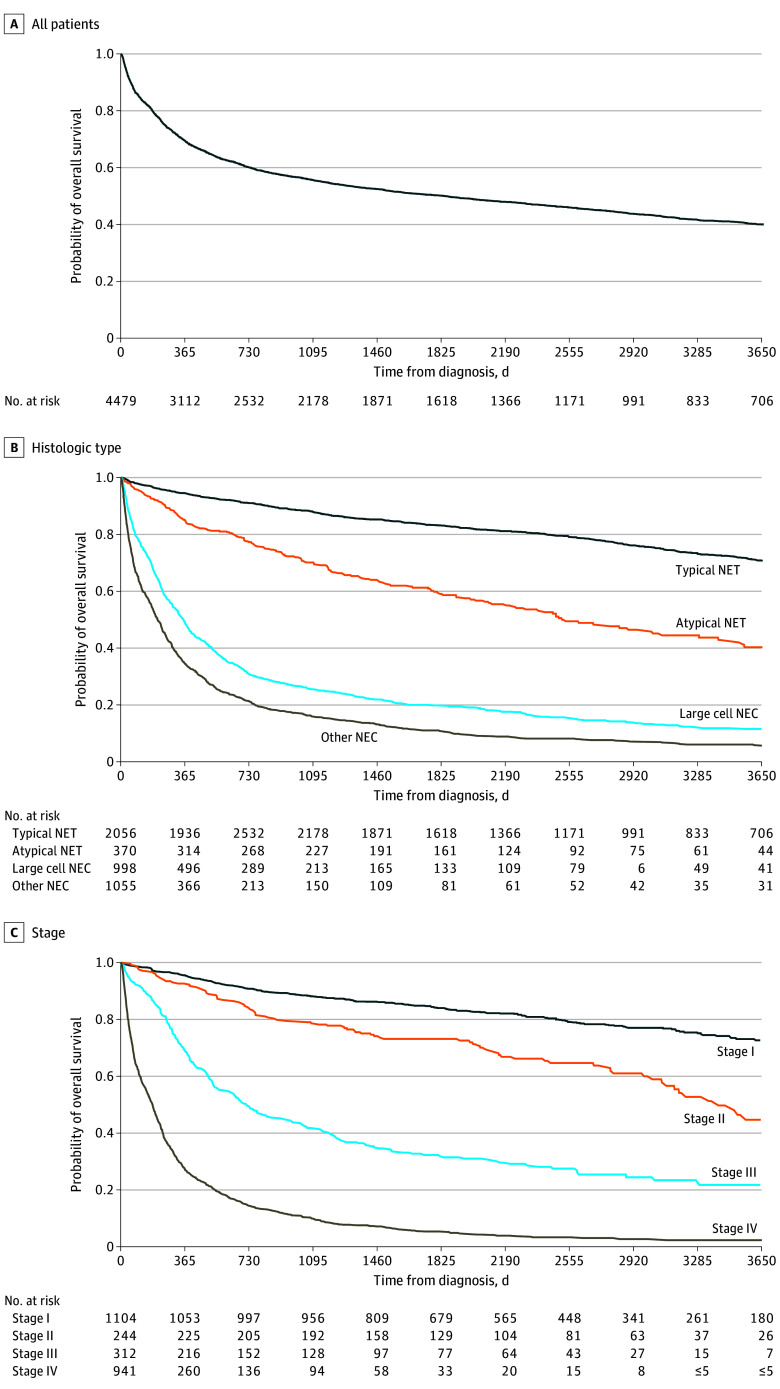
Overall Survival After Lung Neuroendocrine Neoplasm Diagnosis NEC indicates neuroendocrine carcinoma; NET, neuroendocrine tumors.

Estimated OS are presented in Figure 1, eFigure 2 in Supplement 1, and Table 2. OS was 70% (95% CI, 69-71) at 1 year, 50% (95% CI, 49-51) at 5 years, and 40% (95% CI, 39%-41%) at 10 years (Figure 1A). OS was highest for typical NETs with 59% (95% CI, 56%-62%) at 10 years, compared with 46% (95% CI, 42%-50%) for atypical NETs, 20% (95% CI, 18%-22%) for large cell NEC, and 14% (95% CI, 13%-17%) for other NECs (Figure 1B). OS was also highest for stage I disease with 48% (95% CI, 45%-52%) at 10 years, compared with 46% (95% CI, 41%-52%) for stage II, 35% (95% CI, 31%-39%) for stage III, and 16% (95% CI, 13%-19%) for stage IV (Figure 1C).

**Table 2.** Probability of Overall Survival and Lung Cancer–Specific Death After Diagnosis of Lung Neuroendocrine Neoplasm (NEN)

Characteristic	Estimate (95% CI)					
	Overall survival			Lung cancer–specific death (cumulative incidence)		
	1 y	5 y	10 y	1 y	5 y	10 y
All lung NENs	0.70 (0.69-0.71)	0.50 (0.49-0.51)	0.40 (0.39-0.41)	0.27 (0.25-0.28)	0.41 (0.40-0.42)	0.46 (0.45-0.47)
Age, y						
18 to 49	0.86 (0.84-0.88)	0.70 (0.66-0.73)	0.59 (0.55-0.63)	0.15 (0.12-0.18)	0.24 (0.24-0.32)	0.33 (0.29-0.37)
50 to 59	0.76 (0.74-0.78)	0.56 (0.54-0.58)	0.45 (0.43-0.47)	0.23 (0.21-0.25)	0.35 (0.35-0.40)	0.43 (0.40-0.45)
60 to 69	0.72 (0.70-0.73)	0.52 (0.50-0.53)	0.41 (0.39-0.43)	0.25 (0.24-0.27)	0.38 (0.38-0.42)	0.45 (0.43-0.47)
70 to 79	0.66 (0.64-0.67)	0.45 (0.44-0.47)	0.35 (0.33-0.37)	0.29 (0.28-0.31)	0.43 (0.43-0.46)	0.49 (0.47-0.51)
≥80	0.56 (0.54-0.58)	0.36 (0.34-0.38)	0.26 (0.23-0.28)	0.35 (0.32-0.37)	0.47 (0.47-0.51)	0.54 (0.51-0.56)
Sex						
Female	0.72 (0.71-0.73)	0.52 (0.51-0.54)	0.42 (0.41-0.44)	0.24 (0.23-0.26)	0.38 (0.38-0.40)	0.44 (0.43-0.46)
Male	0.67 (0.66-0.69)	0.48 (0.46-0.49)	0.37 (0.36-0.39)	0.29 (0.27-0.30)	0.42 (0.42-0.45)	0.48 (0.47-0.50)
Histology type						
Typical NETs	0.88 (0.87-0.89)	0.72 (0.70-0.74)	0.59 (0.56-0.62)	0.05 (0.05-0.06)	0.12 (0.11-0.14)	0.16 (0.14-0.19)
Atypical NETs	0.82 (0.80-0.84)	0.60 (0.57-0.64)	0.46 (0.42-0.50)	0.15 (0.12-0.17)	0.30 (0.26-0.34)	0.38 (0.33-0.42)
Large cell NECs	0.61 (0.59-0.63)	0.33 (0.31-0.35)	0.20 (0.18-0.22)	0.37 (0.34-0.39)	0.60 (0.57-0.63)	0.68 (0.65-0.71)
Other NECs	0.54 (0.52-0.56)	0.26 (0.24-0.29)	0.14 (0.13-0.17)	0.43 (0.40-0.45)	0.66 (0.63-0.69)	0.74 (0.71-0.77)
Stage at diagnosis						
I	0.85 (0.83-0.88)	0.64 (0.61-0.68)	0.48 **(**0.45**-**0.52)	0.09 (0.11-0.06)	0.20 (0.24-0.16)	0.26 (0.21-0.31)
II	0.84 (0.81-0.87)	0.62 (0.57-0.67)	0.46 **(**0.41**-**0.52)	0.11 (0.14-0.08)	0.25 (0.31-0.20)	0.32 (0.25-0.38)
III	0.76 (0.73-0.79)	0.50 (0.46-0.53)	0.35 **(**0.31**-**0.39)	0.23 (0.26-0.20)	0.44 (0.48-0.41)	0.51 (0.47-0.55)
IV	0.54 (0.52-0.56)	0.28 (0.25-0.31)	0.16 **(**0.13**-**0.19)	0.43 (0.45-0.40)	0.64 (0.67-0.60)	0.70 (0.65-0.7)

Abbreviations: NEC, neuroendocrine carcinoma; NET, neuroendocrine tumors.

**Figure 2.**
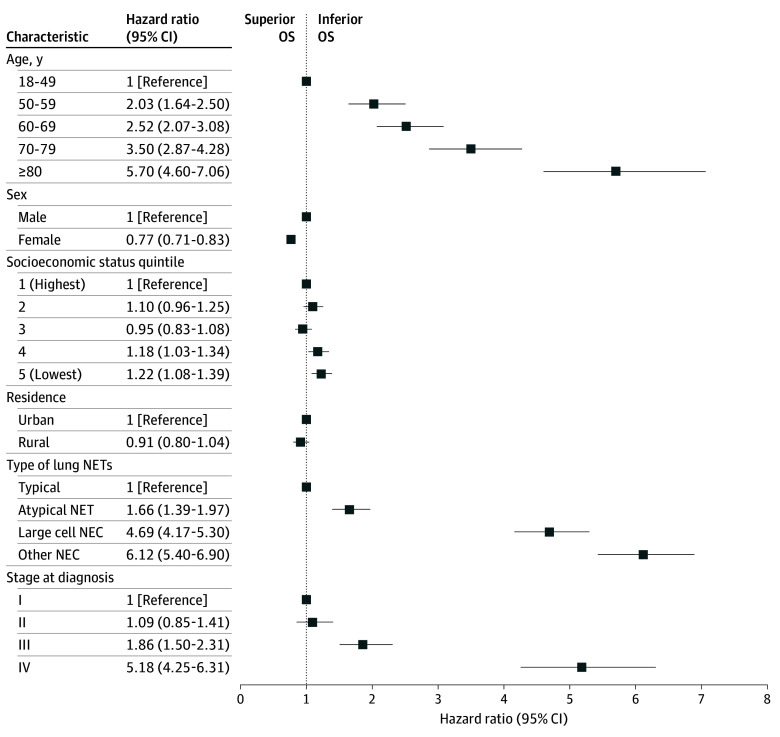
Factors Associated With Overall Survival (OS) and Lung Cancer–Specific Death After Diagnosis of Lung Neuroendocrine Neoplasm Results of Cox multivariable regression for OS and of Fine-Gray multivariable regression for lung cancer–specific death, adjusted for year of diagnosis. Error bars represent 95% CIs. Risk estimates for stage at diagnosis derived from multivariable model restricted to 2010 to 2019 with complete stage data available. NEC indicates neuroendocrine carcinoma; NET, neuroendocrine tumors.

Factors associated with OS are presented in Figure 2. Increasing age, lower socioeconomic status, typical NET, large cell and other NEC, and stages III and IV were all independently associated with inferior OS. Female sex was independently associated with superior OS compared with male.

### LC-Specific Deaths

The cohort for analysis of cause of death included 3946 patients. The median (IQR) follow-up was 43 (9-97) months, during which 1537 (38.9%) died of LC and 746 (18.9%) died of other causes during the follow-up period.

The cumulative incidences of LC-specific death are presented in Table 2, Figure 2, and eFigure 3 in Supplement 1. Among all patients with lung NENs, the cumulative incidence of LC-specific death was 27% (95% CI, 25%-28%) at 1 year, 41% (95% CI, 40%-42%) at 5 years and 46% (95% CI, 45%-47%) at 10 years, which exceeded that of death from other causes at all time points (Figure 2A). The cumulative incidence of LC-specific death increased with advancing age groups and was higher for male than for female participants (eFigure 3 in Supplement 1). It was highest for large cell NECs and other NEC groups (Figure 2) and increased progressively with advancing stage at diagnosis (Figure 2). LC-specific deaths exceeded other causes of death at all time points across most groups except for typical NETs and stage I at diagnosis. For typical NETs, the cumulative incidence of LC death exceeded that of other causes of death up until 3 years after lung NEN diagnosis, after which other causes were the dominant cause of death. For stage I at diagnosis, the cumulative incidence of LC death exceeded that of other causes of death up until 2 year after lung NEN diagnosis, after which it fell below the cumulative incidence of death from other causes.

**Figure 3.**
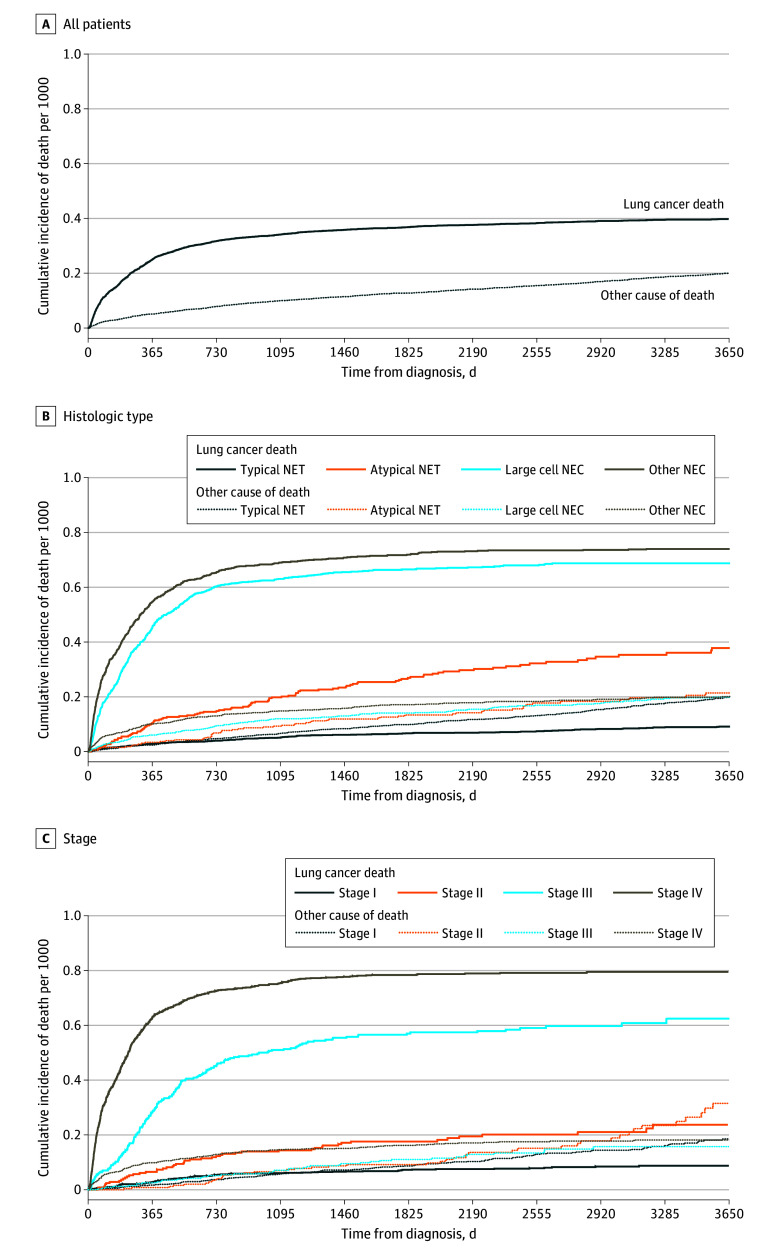
Cumulative Incidence of Lung Cancer–Specific Death and Death From Other Cause After Lung Neuroendocrine Neoplasm Diagnosis NEC indicates neuroendocrine carcinoma; NET, neuroendocrine tumors.

Factors associated with LC-specific death are presented in Figure 3. Advancing age, atypical NET, large cell and other NEC types, and progressive stage were independently associated with higher hazards of LC-specific death. Female sex was independently associated with lower hazards of LC-specific death.

## Discussion

This cohort study observed a 2.87-fold increase in the incidence of lung NENs over 20 years. This appeared to be related mostly to increases in the incidence of typical NETs and stage I disease, by 2.10 and 1.73-fold, respectively. Approximately one-fourth of patients with lung NENs had disseminated (stage IV) disease at the time of diagnosis. Long-term OS was favorable overall, reaching 40% (95% CI, 39%-41%) at 10 years for all lung NENs, but varied by tumor characteristics with better OS identified for typical NETs and for earlier stages at diagnosis. Correspondingly, LC-specific death was favorable, with 46% (95% CI, 45%-47%) at 10 years for all lung NENs. In specific circumstances of typical NET and stage I disease, the risk of death from other causes eventually exceeded the risk of LC-specific death within a few years of diagnosis.

The literature on lung NENs is dominated by studies on diagnostics and histology assessments, followed by examinations of therapy.^18^ Previous studies^1,2,5,19,20,21,22^ using large-scale population-based or registry data from the US, Canada, Norway, and England have provided valuable information on NENs across various primary sites. Very few studies have examined lung-specific epidemiology for NENs. Reports from Iceland (88 cases) and Denmark (347 cases) are available but are hampered by small cohort sizes, reducing their accuracy and generalizability. In the US, the Surveillance, Epidemiology, and End Results (SEER) database has been used to review lung NENs epidemiology.^6^ The long study period (1988-2015) covered changing diagnostic criteria and classification systems and does not reflect contemporary data. In addition, SEER data do not reflect true population-level information and have been known to not represent the US population well.^23^ Most of the trends observed herein echo those from the SEER analysis. However, a higher incidence of typical NETs was observed (1.01 per 100 000 in 2020 herein vs 0.77 per 100 000 in 2015 in SEER). This may be owed to differences in diagnostic practices, detection rates, and access to health care across the 2 regions represented. Of note, the dip in incidence observed in 2020 is thought to be related to the COVID-19 pandemic; drastic drops in cancer diagnosis have been identified that have not been caught up on since.^24,25,26^ The current study is a unique contribution to the existing literature on lung NENs as it offers one of the few detailed epidemiologic analyses of this disease. It used a true population-based design and examined data from a large contemporary cohort over 20 years. Importantly, it is the first we know of to provide detailed estimates of LC-specific death following lung NEN diagnosis.

The observed increase in the incidence of lung NENs can likely be attributed to a combination of more frequent incidental detection and improvements in diagnostic capabilities. Increasing overall use of diagnostic imaging, such as CT scans of the chest, may lead to more incidental lung lesions, and better precision of these imaging modalities may lead to detection of smaller such lesions. In parallel, improvements in diagnostics, including advances in pathology criteria, tumor classification, and characterization of lung tumors with somatostatin-receptor positron emission tomography imaging, have likely contributed to the increased recognition of lung NENs. Refinement of diagnostic criteria, coupled with a heightened awareness of NENs among pathologists and clinicians, likely has made it easier to identify these tumors. In summary, tumors that may have previously remained undiagnosed are now being detected incidentally during imaging for unrelated health concerns and being appropriately identified as NENs. This hypothesis is supported by our observed marked increase in the incidence of typical NETs and stage I tumors. It also aligns with prior hypotheses in NENs epidemiology studies.^1,2^ Documenting the rise in the incidence of lung NENs was important to manage the individual, clinical, and economic impacts of the disease. For instance, increased detection of less aggressive tumors may lead to a greater surveillance burden, and although less common, the care of more aggressive tumors can be resource-consuming for patients, care centers, and health systems alike. This highlights the need for ongoing research and increased awareness among thoracic oncology practitioners to better address lung NENs.

The analysis of OS in this study provided valuable insights into the prognosis of lung NENs. Reported OS for lung NENs in the literature varies widely likely due to differences in therapy, diagnosis, and the predominance of single-center or retrospective studies.^6,18^ The current study provided population estimates with long-term follow-up and minimal loss of information and was able to describe OS up to 10 years after diagnosis, which is crucial for a more indolent malignant neoplasm like NENs. The main factors identified as associated with OS were female sex with better OS and lower socioeconomic status and tumor characteristics with lower OS for more aggressive tumor types and more advanced stage at diagnosis. The association of female sex with superior survival compared with male is particularly interesting. It has been consistently reported in broader NEN epidemiology studies.^1,2,6,22,27,28^

A unique contribution of this study is the detailed analysis of LC-specific deaths, an outcome rarely examined in lung NENs. While cancer-specific mortality has been studied in GEP-NENs, previous research typically aggregates lung NENs with other disease sites and focuses on overall cancer-related death, not LC-specific mortality.^17^ The SEER analysis of lung NENs, while reporting disease-specific survival, did not account for competing risks of death from other causes, an essential factor in assessing cause-specific survival.^29^ LC-specific survival was crucial to examine for lung NENs due to the unique indolent biology of NENs creating longitudinal risk of death from other causes, and stratification by tumor characteristics was important due to their heterogeneity in behavior. Not surprisingly, atypical NETs, NECs, and stage II to IV diseases showed higher risk of death from LC than of other causes throughout postdiagnosis follow-up. However, for typical NETs and stage I disease, death from other causes exceeded LC mortality after 1 and 3 years, respectively. The heterogeneity of these tumor groups likely explains that there were initially more deaths from LC from the minority of more aggressive tumors or treatment-related toxic effects. Overall, this finding highlights the importance of adjusting treatment and surveillance. For instance, higher risk of death from other causes after 1 to 3 years of typical NETs and stage I tumors could support deescalation of surveillance over time. In contrast, sustained LC-specific mortality in atypical NETs, NECs, and stage II to IV tumors may warrant prolonged and more intensive follow-up.

### Limitations and Strengths

This study has limitations. First, it relied on routinely collected data that were not abstracted to specifically address this research question, which introduces potential risks of misclassification and information bias. For example, some prognostic factors, such as lifestyle data or detailed race and ethnicity information, were not available. The use of *ICD-9* codes to identify lung NENs may have led to some misclassification over time, and reliance on pathology diagnoses could underestimate true incidence. Second, the classification of lung NENs used, while based on current standards, may be subject to change as the classification system is being refined.^3,4^ This approach did not include tumor grade databased on Ki67 proliferation index, which is consistent with other studies in this field.^6^ Despite these limitations, the study’s strengths lie in its true population-based design, which allowed for a comprehensive examination of lung NENs incidence and a clinical practice assessment of prognosis.

## Conclusions

This study showed that the incidence of lung NENs has increased 2.87-fold over 20 years in the 21st century, mostly owing to increases in the incidence of typical NETs and stage I tumors. Favorable long-term OS can be achieved after lung NENs, varying by age, sex, and tumor characteristics. In specific circumstances of typical NET and stage I disease, the risk of death from other causes eventually exceeds the risk of LC death within a few years of diagnosis.

## Notes

Supplement 1. eTable 1. Data Sources eTable 2. Strategy to Identify Patients Diagnosed With Lung NENs and Determine Tumor Characteristics eTable 3. Definition of Nonlung Cancer Cause of Death eTable 4. Characteristics of Patients Diagnosed With Lung NENs eFigure 1. Yearly Incidence of Lung NENs Per 100 000 Per Year for All Lung NENs (A), by Patient Sex (B), by Patient Age at Diagnosis (C), by Histology Type (D), and by Stage at Diagnosis (E) eFigure 2. Overall Survival After Lung NEN Diagnosis by Patient Sex (A) and by Patient Age at Diagnosis (B) eFigure 3. Cumulative Incidence of Lung Cancer–Specific Death and Death From Other Cause After Lung NEN Diagnosis by Patient Sex (A) and by Patient Age at Diagnosis (B)

Supplement 2. Data Sharing Statement
