# Supplementary material for: Contemporary Incidence and Survival of Lung Neuroendocrine Neoplasms
Source: JAMA Netw Open. 2025 Oct 2;8(10):e2535125. doi: 10.1001/jamanetworkopen.2025.35125 (PMC12492052; doi:10.1001/jamanetworkopen.2025.35125)
Supplement: Supplement 1. — eTable 1. Data Sources eTable 2. Strategy to Identify Patients Diagnosed With Lung NENs and Determine Tumor Characteristics eTable 3. Definition of Nonlung Cancer Cause of Death eTable 4. Characteristics of Patients Diagnosed With Lung NENs eFigure 1. Yearly Incidence of Lung NENs Per 100 000 Per Year for All Lung NENs (A), by Patient Sex (B), by Patient Age at Diagnosis (C), by Histology Type (D), and by Stage at Diagnosis (E) eFigure 2. Overall Survival After Lung NEN Diagnosis by Patient Sex (A) and by Patient Age at Diagnosis (B) eFigure 3. Cumulative Incidence of Lung Cancer–Specific Death and Death From Other Cause After Lung NEN Diagnosis by Patient Sex (A) and by Patient Age at Diagnosis (B) [file jamanetwopen-e2535125-s001.pdf]

Supplemental Online Content

Hallet JH, Rousseau M, Wakeam E, et al. Contemporary incidence and survival of lung neuroendocrine neoplasms. *JAMA Netw Open*. 2025;8(10):e2535125. doi:10.1001/jamanetworkopen.2025.35125

eTable 1. Data Sources
eTable 2. Strategy to Identify Patients Diagnosed With Lung NENs and Determine Tumor Characteristics
eTable 3. Definition of Nonlung Cancer Cause of Death
eTable 4. Characteristics of Patients Diagnosed With Lung NENs
eFigure 1. Yearly Incidence of Lung NENs Per 100 000 Per Year for All Lung NENs (A), by Patient Sex (B), by Patient Age at Diagnosis (C), by Histology Type (D), and by Stage at Diagnosis (E)
eFigure 2. Overall Survival After Lung NEN Diagnosis by Patient Sex (A) and by Patient Age at Diagnosis (B)
eFigure 3. Cumulative Incidence of Lung Cancer Specific Death and Death From Other Cause After Lung NEN Diagnosis by Patient Sex (A) and by Patient Age at Diagnosis (B)

This supplemental material has been provided by the authors to give readers additional information about their work.

© 2025 Hallet JH et al. *JAMA Network Open*.

eTable 1. Data Sources

Database	Description
**Ontario Cancer Registry (OCR)**	The OCR is a passive, provincial registry all incident cancer diagnoses in Ontario. It includes 96% of cancer diagnoses in the province. Information included in the registry: cancer topography and morphology/histology, and details on diagnosis (e.g., types of contributing information to the diagnosis, dates).
**Activity Level Report (ALR) of Cancer Care Ontario**	Cancer Care Ontario maintains a database of cancers-specific services, including consultations, chemotherapy, and radiotherapy provided by regional cancer centers in the province. Because all radiotherapy is delivered at RCCs, it is a complete source of information. However, because not all cancer patients who receive surgery or chemotherapy visit an RCC, it cannot be used as a population data sources for those treatments.
**Registered Patient Database (RPDB)**	The RPDB is an ICES database derived from all administrative data sources and provides demographic data including age, patient residence, vital status, date of last contact with the healthcare system, and OHIP eligibility.
**Ontario Registrar General (ORG)**	The ORG contains gold standard vital status data for all Ontarians. According to the Vital Statistics Act, it is mandatory to register all deaths occurring in the province.
**Ontario Marginalization Index (ONMarg)**	The ON-MARG is a specialized database using Census data to profile relative area-level marginalization dependency, deprivation, ethnic concentration, and instability at various geographic levels in Ontario.
**Ontario Health Insurance Plan (OHIP)**	The OHIP database contains all physician billing data including information on diagnoses as well as services provided, such as receipt of surgery, chemotherapy, and radiotherapy.
**Ontario Drug Benefit Database (ODB)**	The ODB contains all information for prescription drug claims for individuals covered by the provincial plan (all individuals ≥65 years old), including type of medication, dose, date of prescription filling, and duration of treatment.
**Canadian Institute of Health Information – Discharge Abstract Database and Same-Day Surgery (CIHI-DAD and SDS)**	CIHI-DAD and SDS are mandatory reporting systems that provide information on hospital admissions and same-day surgeries, including diagnoses, procedures, and length of stay.

© 2025 Hallet JH et al. *JAMA Network Open*.

eTable 2. Strategy to Identify Patients Diagnosed With Lung NENs and Determine Tumor Characteristics

**1. Identify neuroendocrine tumors: ICD-O.3**	
81503	Islet cell carcinoma
81501	Islet cell tumor, NOS
81510	Insulinoma, NOS
81513	Insulinoma, malignant
81521	Glucagonoma
81523	Glucagonoma, malignant
815*31*	Gastrinoma
81533	Gastrinoma, malignant
81543	Mixed islet-cell/exocrine adenocarcinoma
81551	VIPoma
81553	VIPoma, malignany
81561	Somatostatinoma
81563	Somastatinoma, Malignant
81571	Enteroglucagonoma
81573	Enteroglucagonoma, malignant
82401	Carcinoid tumor of uncertain malignant potential
82403	Carcinoid tymor, nos
82413	Enterochromaffin-like Cell Carcinoid
82421	Enterochromaffin-like cell tumours, nos
82423	Enterochromaffin-like cell tumours, malignant
82443	Composite carcinoid
82453	Adenocarcinoid
82451	Tubular carcinoid
82463	Neuroendocrine carcinoma
82493	Atypical carcinoid
80133	Large cell neuroendocrine carcinoma of the lung
**2. Restrict to lung primary - t**opography codes, ICD-O.3: C34*	
**3. Classification of lung NEN histology:**	
Atypical neuroendocrine tumor	ICD-O.3: 82493
Large cell carcinoma	ICD-O.3: 80133
Other neuroendocrine carcinoma	ICD-O.3: 80133
Typical neuroendocrine tumor	Any other ICD-O.3

© 2025 Hallet JH et al. *JAMA Network Open*.

eTable 3. Definition of Nonlung Cancer Cause of Death

Category	Non-Cancer Causes of Death	ICD-10 corresponding codes	Cause of death definition
BENIGN TUMORS	In situ, benign or unknown behavior neoplasms	D00-D09	In situ neoplasms
		D10-D36	Benign neoplasms
		D37-D48	Neoplasms of uncertain or unknown behavior
DIABETES	Diabetes Mellitus	E10-E14	Diabetes mellitus
INFECTIOUS DISEASES	Septicemia	A40-A41	Sepsis
	Other Infectious Diseases	A00-A14, A20-39, A42-49, A54-A99, B00-B99	Tuberculosis, syphilis, and other bacterial, viral, and parasitic disease
NON-CARDIAC VASCULAR DISEASES	Hypertension without heart disease	I10	Essential (primary) hypertension
		I12	Hypertensive renal disease
	Cerebrovascular disease	I60-I62	Nontraumatic intracranial haemorrhage
		I63	Cerebral infarction
		I64	Stroke, not specified as haemorrhage or infarction
		I65-I66	Occlusion and stenosis of precerebral/cerebral arteries, not resulting in cerebral infarction
		I67-I69	Other cerebrovascular diseases or Sequelae of cerebrovascular disease
	Atherosclerosis	I70	Atherosclerosis
	Aortic Aneurysm and Dissection	I71	Aortic Aneurysm and Dissection
	Other Diseases of Arteries, Arterioles, Capillaries	I72-I73	Other aneurysm and dissection or other peripheral vascular diseases
		I74	Arterial embolism and thrombosis
		I77	Other disorders of arteries and arterioles
		I78	Diseases of capillaries
RESPIRATORY DISEASES	Pneumonia and Influenza	J09-J18	Influenza and pneumonia
	Chronic obstructive pulmonary disease (COPD)	J40-J42	Bronchitis
		J43	Emphysema
		J44	Other chronic obstructive pulmonary disease
		J45-J46	Asthma or Status asthmaticus
		J47	Bronchiectasis
LIVER AND KIDNEY DISEASES	Chronic Liver Disease and Cirrhosis	K70	Alcoholic liver disease
		K73	Chronic hepatitis
		K74	Fibrosis and cirrhosis of liver
	Nephritis, Nephrotic Syndrome and Nephrosis	N00-N07	Glomerular diseases
		N17-N19	Renal failure
		N25	Disorders resulting from impaired renal tubular function
		N26	Unspecified contracted kidney
		N27	Small kidney of unknown cause
ACCIDENTS AND SELF-HARM	Accidents and Adverse Effects	V01-V99	Transport accidents
		W00-X59	Other external causes of accidental injury
		Y85-Y86	Sequelae of transport accidents or other accidents
		X60-X84	Intentional self-harm

© 2025 Hallet JH et al. *JAMA Network Open*.

	Suicide and Self-Inflicted Injury	Y87	Sequelae of intentional self-harm, assault and events of undetermined intent
ALZHEIMER	Alzheimer's	G30	Alzheimer disease
DISEASES OF THE HEART		I00-I02	Acute rheumatic fever
		I05-I09	Chronic rheumatic heart diseases
		I11	Hypertensive heart disease
		I13	Hypertensive heart and renal disease
		I20-I25	Ischemic heart diseases
		I26-I28	Pulmonary heart disease and diseases of pulmonary circulation
		I30-I32	Diseases of pericardium
		I33	Acute and subacute endocarditis
		I34-I39	Nonrheumatic valve disorders
		I40-I41	Myocarditis
		I42-I43	Cardiomyopathy
		I44-I45	Conduction disorders
		I46	Cardiac arrest
		I47-I49	Arrythmias
		I50	Heart failure
		I51	Complications and ill-defined descriptions of heart disease
OTHER NON-CANCER	Symptoms, Signs and Ill-Defined Conditions	R00-R99	Symptoms, signs, abnormal results of clinical or other investigative procedures, and ill-defined conditions regarding which no diagnosis classifiable elsewhere is recorded.
	Other Cause of Death	All others not included in any of the above	
CANCER DEATH – NOT LUNG CANCER	Malignant neoplasms	ICD-9 140-161 and 163-239	

© 2025 Hallet JH et al. *JAMA Network Open*.

eTable 4. Characteristics of Patients Diagnosed With Lung NENs

		**All patients (n=4,479)**
**Age at diagnosis** (years old) – median (IQR)		67 (57-74)
**Sex**	Female	2,521 (56.3%)
	Male	1,958 (43.7%)
**Rural residence**	Urban	4,025 (89.9%)
	Rural	447 (10.0%)
**Socioeconomic status** (SES)	1st quintile (highest SES)	800 (17.9%)
	2nd quintile	851 (19.0%)
	3rd quintile	871 (19.4%)
	4th quintile	912 (20.4%)
	5th quintile (lowest SES)	994 (22.2%)
**High comorbidity burden**		357 (8.0%)
**Histology type**	Typical NET	2,056 (45.9%)
	Atypical NET	370 (8.3%)
	Large cell NEC	998 (22.3%)
	Other NEC	1,055 (23.6%)
**Stage at diagnosis*** (n=2,976)	I	1,202 (26.8%)
	II	282 (6.3%)
	II	389 (8.7%)
	IV	1,103 (24.6%)
	Missing	1,503 (33.6%)
**Prior other cancer diagnosis**		724 (16.2%)

Values are n(%) unless otherwise specified
* data restricted to 2010-2019 due to availability
IQR, inter-quartile range; SES, socioeconomic status; NET, neuroendocrine tumor; NEC, neuroendocrine carcinom

© 2025 Hallet JH et al. *JAMA Network Open*.

eFigure 1. Yearly Incidence of Lung NENs Per 100 000 Per Year for All Lung NENs (A), by Patient Sex (B), by Patient Age at Diagnosis (C), by Histology Type (D), and by Stage at Diagnosis (E).

A

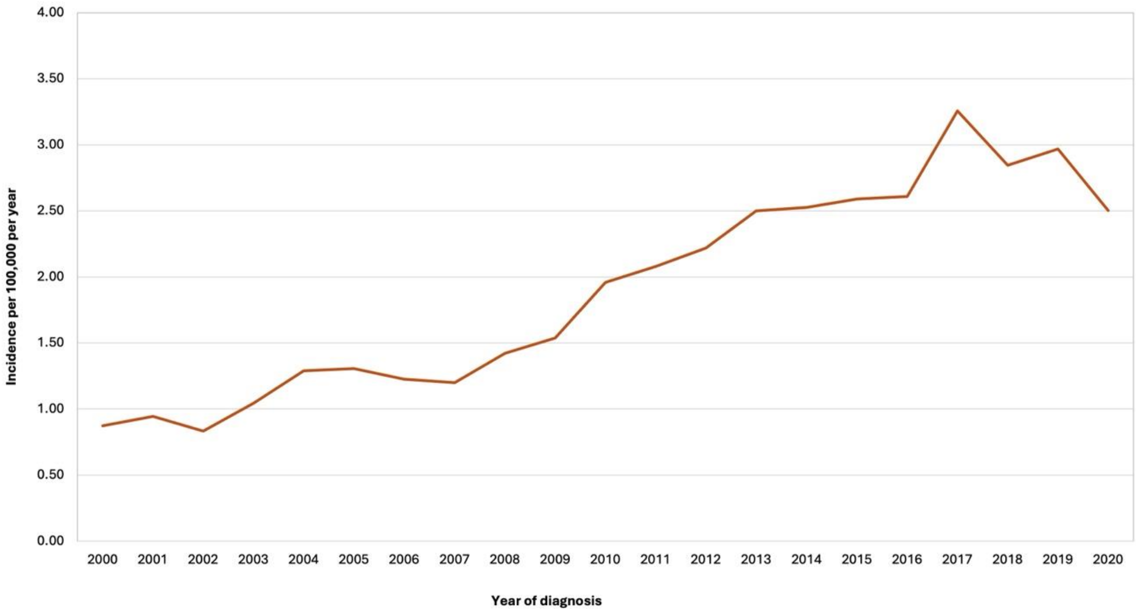

B

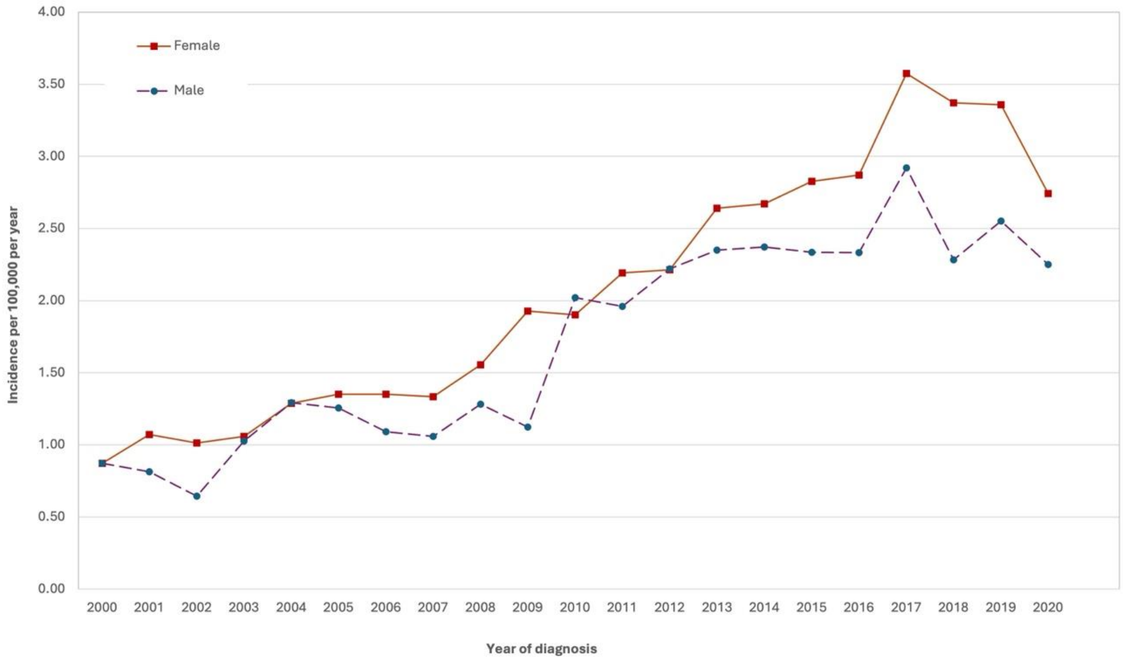

© 2025 Hallet JH et al. *JAMA Network Open*.

C

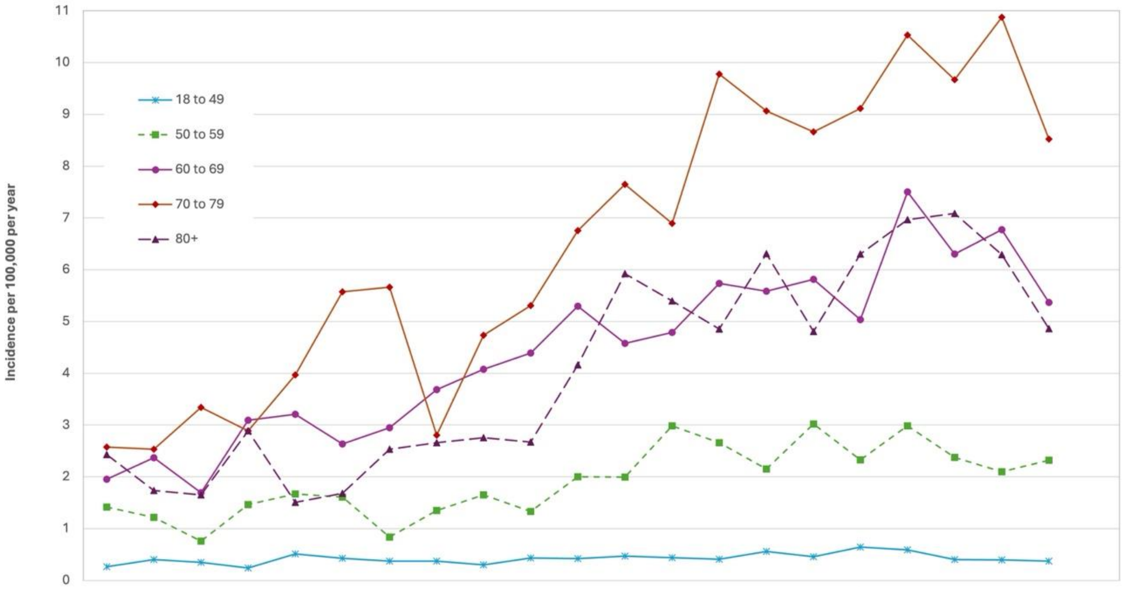

D

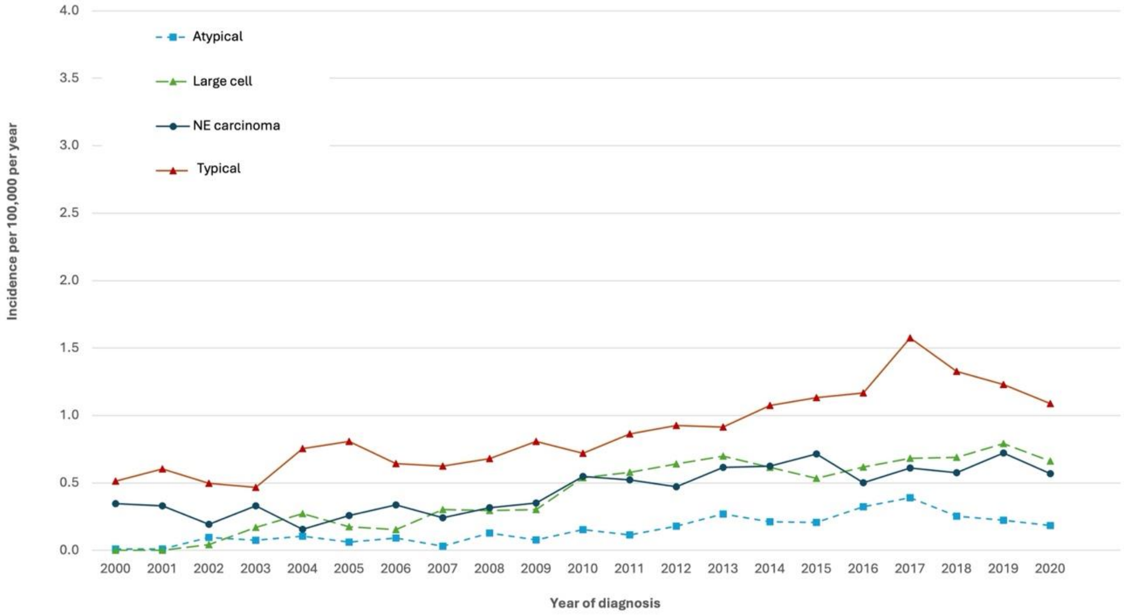

© 2025 Hallet JH et al. *JAMA Network Open*.

E

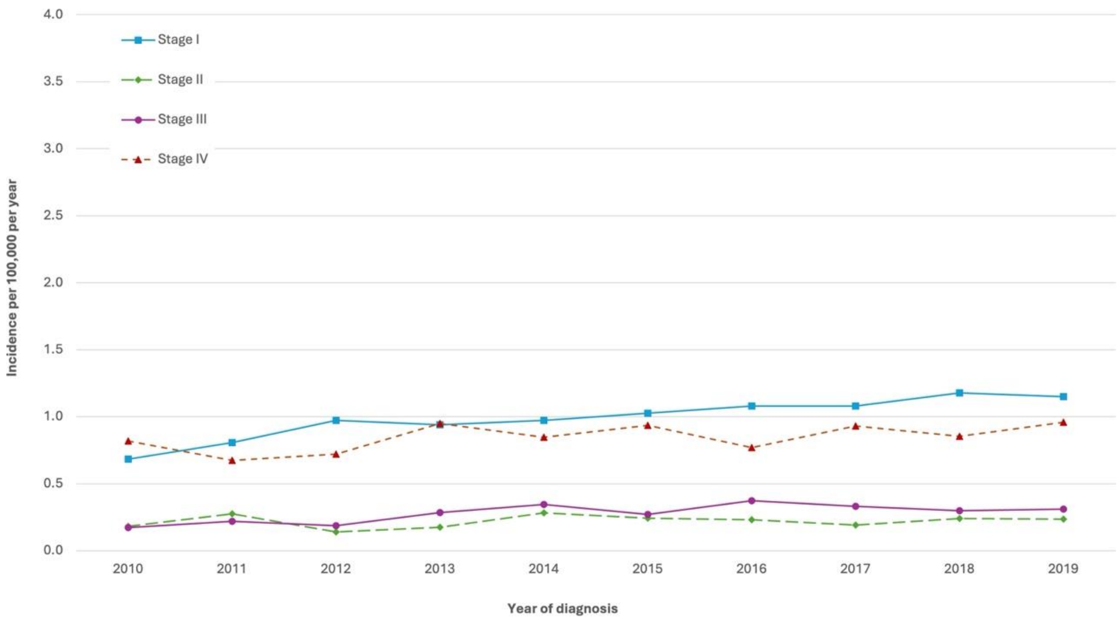

© 2025 Hallet JH et al. *JAMA Network Open*.

eFigure 2. Overall Survival After Lung NEN Diagnosis by Patient Sex (A) and by Patient Age at Diagnosis (B).

Solid lines represent the cumulative incidence of lung cancer death and dotted lines represent the cumulative incidence of death from other causes.

A

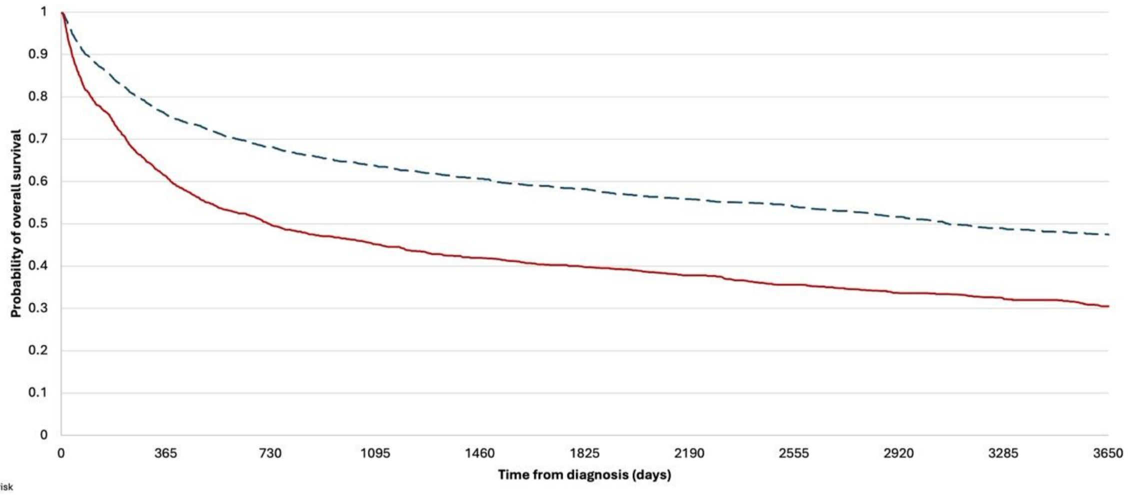

Numbers at risk

Female	2,521	1.914	1,606	1,400	1,13	1,039	886	769	643	530	456
Male	1,958	1,198	926	778	658	579	480	403	348	303	250

B

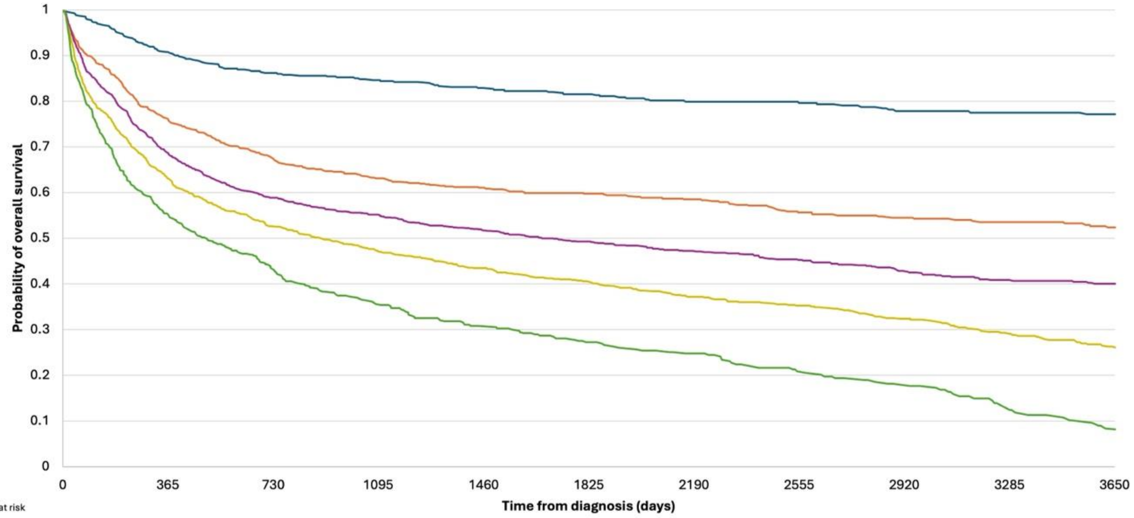

Numbers at risk

Age											
18-49	533	480	442	413	83	355	319	288	260	231	214
50-59	789	600	507	437	95	354	310	265	228	202	178
60-69	1,375	946	756	657	552	465	390	333	276	228	191
70-79	1,286	811	625	515	423	349	70	28	184	145	108
80+	496	75	202	156	118	95	77	57	43	27	15

© 2025 Hallet JH et al. *JAMA Network Open*.

eFigure 3. Cumulative Incidence of Lung Cancer Specific Death and Death From Other Cause After Lung NEN Diagnosis by Patient Sex (A) and by Patient Age at Diagnosis (B).
Solid lines represent the cumulative incidence of lung cancer death and dotted lines represent the cumulative incidence of death from other causes.

A

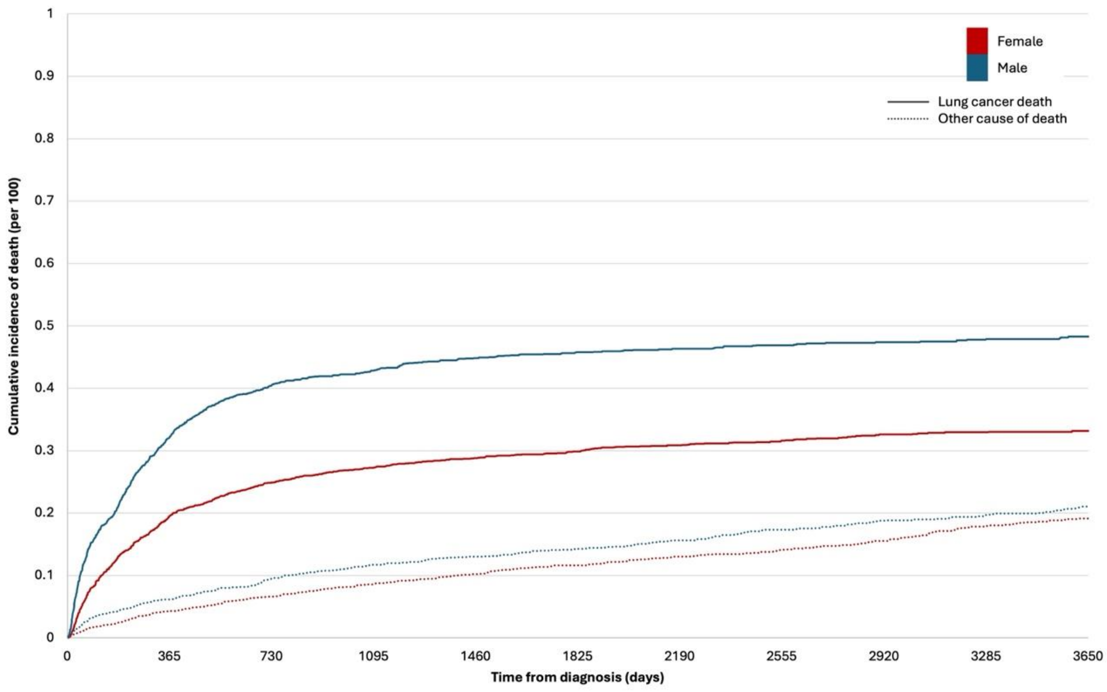

B

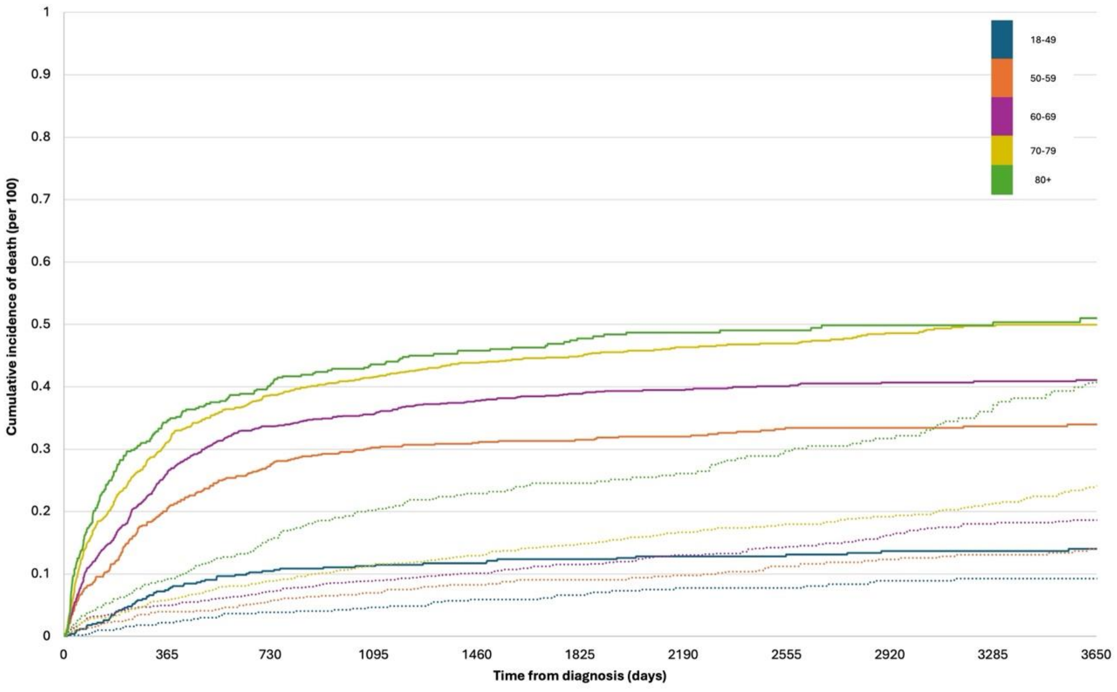

© 2025 Hallet JH et al. *JAMA Network Open*.
