# Supplementary material for: Contemporary Incidence and Survival of Lung Neuroendocrine Neoplasms
Source: JAMA Netw Open. 2025 Oct 2;8(10):e2535125. doi: 10.1001/jamanetworkopen.2025.35125 (PMC12492052; doi:10.1001/jamanetworkopen.2025.35125)
Supplement: Supplement 2. — Data Sharing Statement [file jamanetwopen-e2535125-s002.pdf]

Data Sharing Statement

Hallet. Contemporary Incidence and Survival of Lung Neuroendocrine Neoplasms. *JAMA Netw Open*. Published October 02, 2025. doi:10.1001/jamanetworkopen.2025.35125

Data
Data available: No

Additional Information
Explanation for why data not available: The datasets from this study are held securely in coded form at ICES. While data sharing agreements prohibit ICES from making the datasets publicly available, access may be granted to those who meet pre-specified criteria for confidential access, available at www.ices.on.ca/DAS. The dataset creation plan and underlying analytic code are available from the authors upon reasonable request, understanding that the computer programs may rely upon coding templates or macros that are unique to ICES and are therefore either inaccessible or may require modification.
